# The effect of saffron (*Crocus sativus* L.) supplementation on blood pressure, and renal and liver function in patients with type 2 diabetes mellitus: A double-blinded, randomized clinical trial

**Published:** 2019

**Authors:** Fatemeh Ebrahimi, Naheed Aryaeian, Naseh Pahlavani, Davood Abbasi, Agha Fatemeh Hosseini, Soudabeh Fallah, Nariman Moradi, Iraj Heydari

**Affiliations:** 1 *Skull Base Research Center, Loghman Hakim Hospital, Shahid Beheshti University of Medical Sciences, Tehran, Iran.*; 2 *Department of Nutrition, School of Public Health, Iran University of Medical Sciences, Tehran, Iran.*; 3 *Department of Nutrition, Faculty of Medicine, Mashhad University of Medical Sciences, Mashhad, Iran.*; 4 *Faculty of Medicine, Iran University of Medical Sciences, Tehran, Iran.*; 5 *Department of Biostatistics, School of Public Health, Iran University of Medical Sciences, Tehran, Iran.*; 6 *Department of Clinical Biochemistry, Faculty of Medicine, Iran University of Medical Sciences, Tehran, Iran.*; 7 *Department of Clinical Biochemistry, Faculty of Medicine, Kurdistan University of Medical Sciences, Sanandaj, Iran.*; 8 *Department of Endocrinology, School of Medicine, Iran University of Medical Sciences, Tehran, Iran.*

**Keywords:** Crocus sativus, Diabetes mellitus type 2, Herbal medicine, Blood pressure, Nephropathy

## Abstract

**Objective:**

Microalbuminuria and hypertension are the risk factors for diabetic nephropathy, and increased levels of liver enzymes are prevalent among diabetic patients. The aim of this research was to examine the effects of *Crocus sativus* supplementation on nephropathy indices, liver enzymes, and blood pressure in patients with type 2 diabetes (T2D).

**Materials and Methods:**

This placebo-controlled, randomized clinical trial was performed among 80 T2D patients. Subjects were randomly assigned to either *Crocus sativus* (n = 40) or placebo (n = 40) groups and treated with *C. sativus* and or placebo for 12 weeks, respectively. Alkaline phosphatase (ALP), aspartate aminotransferase (AST), alanine aminotransferase (ALT), serum urea, creatinine, 24-hr urine albumin, systolic blood pressure (SBP), diastolic blood pressure (DBP), physical activity, and dietary intakes were measured and blood samples were taken at baseline and after the 12‑week intervention to assess the differences between the two groups.

**Results:**

*C. sativus* supplementation compared with the placebo resulted in a significant reduction of SBP (P<0.005). However, changes in other indices including liver enzymes, serum creatinine, serum urea, and 24-hr urine albumin, and DBP were not significantly different between the two groups (p>0.05). Also, no significant changes in dietary intakes and physical activity were seen between the two groups.

**Conclusion:**

This report shows that daily supplementation with 100 mg *C. sativus* powder improved SBP. However, it did not considerably improve DBP, nephropathy indices and liver functions in T2D patients after 12 weeks of administration.

## Introduction

Diabetes mellitus is a metabolic disorder which is related with severe hyperglycemia, impaired metabolism of carbohydrates, and fats, as well as proteins that give rise to relative or absolute insulin deficiency or insulin resistance that is divided into two major classes, type 1 and 2. 90% of the total prevalence of diabetes is type 2 diabetes (Larejani and Zahedi, 2001 ▶; Li et al., 2012 ▶). The prevalence of T2D is 6.4% in adults worldwide and it is anticipated that it reaches 4.4% in all groups in 2030 (Elshater et al., 2009 ▶; Shanmugam et al., 2009 ▶). The prevalence of diabetes according to the Esteghamati et al. study was 7.7% equal to 2 million cases when extrapolated to the Iranian population within the age range 25–64 years (Esteghamati et al., 2008 ▶).

Because of hyperglycemia in T2D, liver disorder including fatty liver disease is also very rampant in the diabetic population (Lavie et al., 2003 ▶). Resistance to insulin in patients with T2D has also been established as a predisposing factor of increased levels of liver enzymes such as ALT, ALP, AST, dyslipidemia, hypertension, and cardiovascular diseases (Esteghamati et al., 2008 ▶; Jyothirmayi and Kumar, 2011 ▶). Elevated oxidative stress and hypertension in diabetic patients may lead to some complications including retinopathy and nephropathy (Darko et al., 2002 ▶).

Hypertension and microalbuminuria are the risk factors for diabetic nephropathy that is observed in nearly 40 percent of all individuals with T2D and can lead to end-stage kidney disease (Parving et al., 2001 ▶). 

Lack of proper control of diabetes complications (e.g. micro- and macro-vascular) causes a range of problems that diminish the life's quality, inflict high expenses to the healthcare system and increase mortality rate (Whiting et al., 2011 ▶). Therefore, it is very important for the health care systems to adopt appropriate strategies to reduce the complications of diabetes. In addition to the usual treatments for T2D such as insulin therapy and blood glucose lowering drugs, complementary and herbal medicine therapies have useful effects and ameliorate some risk factors in diabetic subjects (Azadmehr et al., 2014 ▶). Several clinical trials have shown that herbal medicine has positive effects on diabetes complications (Moradabadi et al., 2013 ▶). However, there is no convincing evidence for using herbal and complementary in previous works (Mirfeizi et al., 2016 ▶). 

Saffron or *Crocus sativus *L. is a bulbous perennial of the iris family (Iridaceae) valued for its golden-colored, pungent stigmas, which are dried and used to color and flavor foods as well as a dye (Jelodar et al., 2018 ▶; Zheng et al., 2005 ▶). *Crocus sativus* contains crocetin that is effective in the control and treating some conditions such as dyslipidemia and hypertension (Xi et al., 2007 ▶). The possible lowering effect of *C. sativus* on blood pressure is related to some of its components include crocin, picrocrocin, safranal, and crocetin (Imenshahidi et al., 2010 ▶; Kaur and Khanna, 2012 ▶). Previous studies showed that *C. sativus* may protect the liver and kidneys against some toxic agents (Huang et al., 2014 ▶; Omidi et al., 2014 ▶; Tajadadi-Ebrahimi et al., 2014 ▶).

Although some reports indicated the effect of *C. sativus* on the metabolic indices, there is no complete documentation about the useful effects of *C. sativus* supplementation on T2D complications. The objective of our study was to determine the effects of *C. sativus* supplementation compared with placebo, on liver enzymes (Alkaline phosphatase (ALP), aspartate aminotransferase (AST) and alanine aminotransferase (ALT)), nephropathy indices (serum urea, serum creatinine, and 24-hr urine albumin), systolic blood pressure (SBP) and diastolic blood pressure (DBP) in patients with T2D. 

## Materials and Methods

**Study design**

We conducted a prospective, double-blind, placebo-controlled, randomized study that was registered in the Iranian Registry of Clinical Trials (IRCT, www.irct.ir) under registration No. IRCT201510259472N9. The study was approved by the Ethics Committee of Iran University of Medical Sciences, Tehran, Iran (registration No: IR.IUMS.REC.1394.26583), and a written informed consent was obtained from all subjects. Trial registration, baseline/eligibility testing, allocation, and follow-up were all conducted in accordance with Consolidated Standards of Reporting Trials guidelines (Schulz et al., 2010 ▶).

**Participants**

Ninety patients aged 30–70 years with T2D were recruited between December 2015 and October 2017.

The understudy subjects were diagnosed with T2D by an endocrinologist on the basis of the results of the hematologic tests and met the criteria required for being enrolled in this research work. These criteria included having a disease duration of at least 3 years, an HbA1c level of 6.5-10%, and a body mass index (BMI) of 20-35 kg/m^2^. Exclusion criteria were insulin therapy at baseline or during the study, changes in the type or dose of medications, changes in diet or daily physical activity, taking nutritional supplements during the last 3 months, smoking, and alcohol abuse during the last year, any acute illnesses or some chronic diseases including kidney, liver, cardiovascular, and gastrointestinal diseases, pregnancy and lactation, consumption of *C. sativus* or other botanical supplements, *C. sativus* hypersensitivity, and consumption of less than 80% of supplements during the study period.

We used the formula suggested for estimation of the sample size required for a randomized clinical trial ($n=\frac{{2\left(Z1+\mathrm{Z2}\right)\sigma }^{2}}{d^{2}}$ ). The sample size was calculated 45 subjects in each group, considering a study power of 80%, type I error of 5% (α=0.05), type II error of 20% (β=0.20), and alanine aminotransferase (ALT) as a key variable. Totally, 90 individuals were selected at the baseline, and were followed for 12 weeks. Subjects were randomly allocated to either *C. sativus* supplemented group (n=45) or placebo-treated group (n=45). The randomization scheme was generated using a computer-based random-number generator.

**Figure 1:**
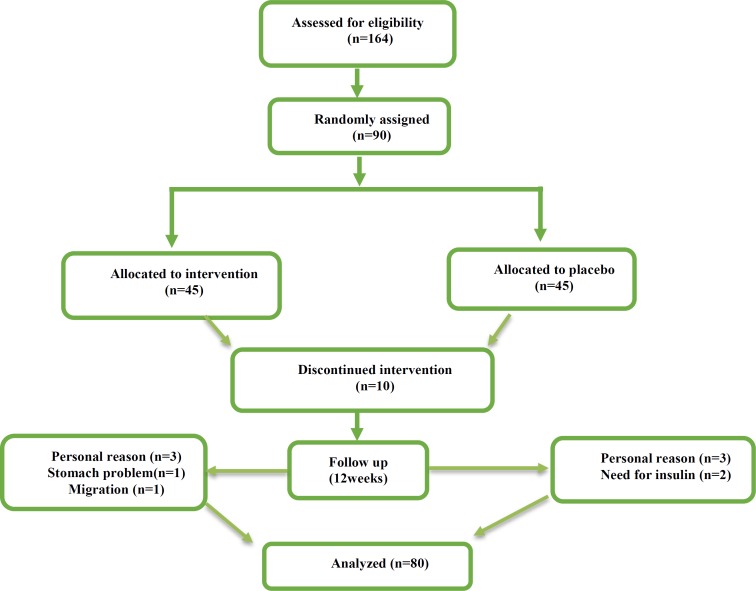
Schematic diagram of the study; individuals in the intervention group received powder containing 100 mg of saffron per day during the study; those in the placebo group received 100 mg placebo (maltodextrin) powder at the same times

Although 90 T2D patients were deemed eligible for inclusion in the study after an initial visit, only 80 completed the study (five individuals in the saffron group either withdrew because of personal reasons, stomach problems or were lost to follow-up because they migrated elsewhere and five individuals in control group, due to personal reasons and need for insulin therapy). Of these, 40 were randomized to the saffron group and 40 were randomized to the control group (Figure 1). In this trial, 100% of powders were consumed in both groups and the rate of compliance in our study was high. 

***C. sativus*** **preparation and dose of intervention**

*Crocus sativus* was supplied by the Novin Saffron Co. (Mashhad, Iran). It was formulated as a dried powder of *C. sativus* in the Faculty of Pharmacy at Tehran University of Medical Sciences. Placebo powder (maltodextrin) matched saffron powder in size and volume of content and was manufactured by the same company.

Different doses of *Crocus sativus* have been used in different studies (from 20 to 400 mg/day), and we selected a dose of 100 mg/day which is a safe dose for intervention (Ayatollahi et al., 2014 ▶; Broadhead et al., 2019 ▶; Moazen-Zadeh et al., 2018 ▶; Shemshian et al., 2014 ▶).

**Measurements**

Detailed information about sex, age, anthropometric indices, medical history, taking medical drugs, and supplements use were collected. Anthropometric measurements of weight, height, and BMI were recorded for each subject at the outset and end of the study. We measured weights without shoes and with minimal clothes to the nearest 0.5 kg. Height was measured by using a non-stretched tape measure without shoes, while the shoulders were in a normal position. BMI was determined as weight in kilograms divided by height in meters squared.

At the beginning and end of the study, physical activity level was determined by using International Physical Activity Questionnaire (IPAQ), for which reliability and validity were evaluated in Iran (Vasheghani-Farahani et al., 2011 ▶). This questionnaire assesses walking time, moderate and vigorous intensity physical activities, and the amount of time spent sitting in a typical week. We asked all participants not to change the physical activity during the research time. However, the differences between the two groups in physical activity were adjusted in the ultimate analysis.

All individuals were permitted to take their regular medications based on their physician᾽s prescription. We obtained 3-day dietary records at the beginning, in the middle and at the end of the study. The mean values from all three time points were considered regular dietary intake of participants during the intervention period. Participants were asked to record everything they take during the day, including the between-meal, supplements, and late-evening snacks as accurately as possible. For dietary records, we used values in household measurements (Ghaffarpour et al., 1999 ▶).

To obtain macro and micronutrient consumption of the individuals according to dietary records, we used Nutritionist IV software (based on US National Nutrient Databank) modified for Iranian foods. The reliability and validity of 3-day food record for Iranian adults had been confirmed previously (Mohammadifard et al., 2015 ▶). 

Blood samples (10 ml) were taken in 10-12 hours fasting state at the beginning and after 12 weeks of intervention. The serum was obtained by high-speed centrifugation (3000 rpm) at 4°C for 10 min and frozen immediately at −80°C until assay. 

Serum urea, serum creatinine, 24-hr urine albumin, ALT, AST, and ALP were measured using Hitachi 911 automatic analyzer (Hitachi Ltd, Tokyo, Japan). SBP and DBP were measured three times in every session after a 15-min rest sitting down by the mercury sphygmomanometer (Zyklusmed Co, Germany).

**Intervention**

After assignment of subjects to *C. sativus* or placebo group, participants in the *C. sativus* group received daily a powder containing 100 mg *C. sativus* in the morning, and those in the placebo group daily received the same amount of placebo (maltodextrin) for 12 weeks. According to previous studies, maltodextrin at a dose of 100 mg/day has no effect on cardiovascular and liver functions (Hofman et al., 2016 ▶). The appearance of the placebo powders (including color and packaging) was similar to the *C. sativus* powder. All subjects were recommended not to change the common dietary intake during the study, and not to take any supplements (vitamins, minerals, proteins carbohydrates, etc.).

**Statistical analysis**

We analyzed all data by means of SPSS software version 18 (SPSS, Inc., Chicago, IL, USA). We used the Kolmogorov–Smirnov test to examine the normal distribution of variables. Log transformation was conducted for non-normally distributed variables. Independent-samples t-test was applied to examine the homogeneity of general characteristics, anthropometric measurements, and dietary intakes in two groups. We used paired samples t-test (Wilcoxon in non-parametric distribution) to assess the effects of both *C. sativus* and placebo supplements on nephropathy indices, hepatic enzymes, and blood pressure measurements. 

 To compare the changes between groups, we applied independent-samples t-test (Mann-Whitney U test in case of non-parametric distribution). We used a chi-squared test for physical activity analyses. To find if the magnitude of the change depends on baseline values, physical activity, and usual dietary intake, we adjusted these variables by using analysis of covariance (ANCOVA) to obtain the independent effect of *C. sativus* supplementation on liver enzymes, blood pressure, and nephropathy indices. A p<0.05 was considered significant. All values are reported as mean±SD.

## Results

The baseline data of subjects are shown in Table 1. There was no statistically significant difference in sex, age, weight, height, waist circumference, BMI, disease duration, and type of consumed oral hypoglycemic drugs between the two groups.

The usual dietary intake of participants throughout the intervention on the basis of 3-day dietary records is presented in Table 2. No significant differences were found in usual intake of energy, protein, carbohydrate, and fat between *C. sativus *and placebo groups.

**Table 1:** General baseline characteristics of subjects who received either *C. sativus* or placebo

Variables		*Crocus sativus* group^a^ (n=40)	Placebo group^b^ (n=40)	p-value^c^
Age (year)		55.2±7.3	53±10.6	0.61
Sex	Male n(%)	20 (50)	16 (40)	0.51
	Female n(%)	20 (50)	24 (60)	
Height (cm)		162±9.9	163.7±8.3	0.41
Weight (kg)		75.3±12.8	80.3±12.8	0.08
BMI (kg/m^2^)		29.3±4.9	30.5±4.7	0.25
WC (cm)		106±9.5	107.8±12.9	0.75
Duration of T2D (year)		7.8±5.4	6.6±6.1	0.13
Types of hypoglycemic drugs	Metformin n(%)	4 (10)	6 (15)	0.454
	Metformin+ glibenclamide n (%)	8 (20)	13 (32.5)	
	Metformin+ glibenclamide n(%)	10 (25)	8 (20)	
	Other drugs n (%)	18 (45)	13 (32.5)	

Abbreviations: BMI, Body Mass Index; WC, Waist Circumference. All quantitative values are expressed as means±SDs. All qualitative values are expressed as numbers and percentages (%).

a Received 100 mg *Crocus sativus* per day during the study.

b Received 100 mg placebo per day during the study.

c Obtained from independent-samples t test for quantitative values and Chi-square for qualitative values.

**Table 2:** Usual dietary intake of participants who received either *C. sativus* or placebo during the study

Variables	*Crocus sativus* group^a^ (n=40)	Placebo group^b^ (n=40)	p-value^c^
Energy Intake (Kcal)	1848±164	1890±160	0.608
Carbohydrate (g per day)	230.6±38.8	279.4±277.7	0.275
Protein Intake (g per day)	59.4±12.5	80.2±158.5	0.411
Fat (g per day)	83.2±14.2	84.2±17.2	0.799

All values are presented as mean±SD.

a Received 100 mg *C. sativus* per day during study.

b Received 100 mg placebo per day during the study.

c As evaluated by independent-samples t test.

Moreover, physical activity level was not different before and after the study (p>0.05) (Table 3).

Baseline and post-intervention values of ALT, AST and ALP are presented in Table 4. In this study, supplementation with 100 mg *C. sativus* per day compared with placebo (100 mg maltodextrin) for 12 weeks had no effect on liver enzymes (p>0.05). 

**Table 3:** Physical activity measurements at the baseline and 12 weeks after intervention in the two groups

p-value b	Control Group (n=40)			*Crocus sativus* Group (n=40)			Group
	Severe	Moderate	Low	Severe	Moderate	Low	Intensity of PA a Stage of Intervention
**0.124**	3 (7.5)	5 (12.5)	32 (80)	4 (10)	12 (30)	24 (60)	**Before Intervention ** **n (%)**
**0.124**	3 (7.5)	5 (12.5)	32 (80)	4 (10)	12 (30)	24 (60)	**After Intervention** **n (%)**

a . Physical activity

b . As assessed by Chi-squared test

**Table 4:** liver functional tests measurements at baseline and 12 weeks after the intervention in subjects who received either *Crocus sativus *or placebo

Variables	Crocus sativus group^a^ (n=40)		p-value^b^	Placebo group^c^ (n=40)		P-valueb	p-value^d^
	Baseline	After		Baseline	After		
AST (U/L)	22.4±10.3	21.2±8.8	0.248	22.1±8.5	19.8±7.5	0.02	0.448
ALT (U/L)	22.35±10.7	21±11.9	0/308	23.6±11.1	20.8±11	0.046	0.438
ALP (U/L)	177.1±49.6	172.3±45.8	0.419	167.1±46.6	166.4±45.2	0.871	0.582

Abbreviations: AST, Aspartate Aminotransferase; ALT, Alanine Aminotransferase; ALP, Alkaline phosphatase. All values are presented as means±SD.

a Received 100 mg *C. sativus* per day during study.

b As assessed by paired-samples t test.

c Received 100 mg placebo per day during the study.

d As assessed by independent-samples t test.

Intake of *C. sativus* compared with placebo did not significantly affect nephropathy indices including serum urea, serum creatinine, 24-hr urine albumin, and DBP (Table 5). Consumption of *C. sativus*, compared with placebo, resulted in a significant decrease in SBP (P=0.005) (Table 5).

In addition, analysis following adjustment of baseline values, physical activity and usual dietary intake throughout the study revealed no significant changes in AST, ALT, ALP, DBP, serum creatinine, serum urea, and 24-hr urine albumin (p>0.05), however, changes in SBP were significant (p<0.05) (Table 6).

**Table 5:** Blood pressure and nephropathy indices measured at baseline and 12 weeks after the intervention in subjects who received either *Crocus sativus* or placebo

Variables	Crocus sativus group^a^ (n=40)		p-value^b^	Placebo group^c^ (n=40)		p-value^b^	p-value^d^
	Baseline	After		Baseline	After		
SBP (mmHg)	132.7±21.3	124.5±13.2	0.004	127.4±15.3	128.3±12.4	0.604	0.005
DBP (mmHg)	79.5±10.8	76.7±9.9	0.104	79.7±11.1	75.9±14	0.091	0.621
Serum creatinine (mg/dl)	1.09±0.17	1.1±0.2	0.430	1.1±0.19	1.1±0.2	0.964	0.505
Serum urea (mg/dl)	32.6±8.6	30.4±8.5	0.067	31.4±7.4	31.2±7.7	0.817	0.24
	Baseline	After	p-value^f^	Baseline	After	p-value^f^	p-value^e^
Albumin (mg/24 hr)	38.4±22.8	35.7±26.7	0.332	29.8±26.7	29.9±25.4	0.828	0.401

Abbreviations: SBP, Systolic blood pressure; DBP, Diastolic blood pressure.

a Received 100 mg *C. sativus* per day during the study.

b As assessed by paired-samples t test.

c Received 100 mg placebo per day during the study.

d As assessed by independent-samples t test.

f As assessed by Wilcoxon signed rank test.

e As assessed by Mann-Whithney U test.

**Table 6:** Adjusted changes in the liver functional tests, blood pressure and nephropathy indices measurement in subjects who received either *Crocus sativus *or placebo

Variables	*Crocus sativus* group^a^ (n=40)	Placebo group^b^ (n=40)	p-value^c^
AST (U/L)	-1.2±6.6	-2.3±5.9	0.326
ALT (U/L)	-1.3±8.3	-2.8±8.6	0.522
ALP (U/L)	-4.8±37.1	-0/7±28.1	0.858
SBP (mmHg)	-7.2±14.6	0.8±9.9	0.006
DBP (mmHg)	-2.7±10.3	-3.8±10.2	0.631
Serum creatinine (mg/dl)	0.02±0.18	-0.001±0.13	0.583
Serum urea (mg/dl)	-2.25±7.54	-0.27±7.3	0.334
Albumin (mg/24 hr)	-0.65±7.01	0.05±5.8	0.585

Abbreviations: AST, Aspartate Aminotransferase; ALT, Alanine Aminotransferase; ALP, Alkaline phosphatase; SBP, systolic blood pressure; DBP, diastolic blood pressure. All values are expressed as means±SD.

a Received 100 mg *C. sativus* per day during study.

b Received 100 mg placebo per day during the study.

c As assessed by AVCOVA test.

## Discussion

To the best of our knowledge, this study is one of few studies that examined the effect of *C. sativus* supplementation on blood pressure, nephropathy indices and liver enzymes levels in patients with T2D. The results of the present study indicated that 12-week supplementation with 100 mg daily of *C. sativus* significantly decreases SBP in T2D patients compared with baseline and placebo group. This effect remained significant even after adjustment of primary values, physical activity and usual dietary intake of subjects throughout the study. We did not find any significant effect for *C. sativus* on serum AST, ALT, and ALP levels as well as DBP, serum urea and creatinine, and 24-hr urine albumin. Patients with T2D are very susceptible to nephropathy, increased activity of liver enzymes, and elevated blood pressure. Hypertension and increased liver enzymes among patient with T2D can lead to vascular disease and insulin resistance (Beckman et al., 2002 ▶; Hanley et al., 2007 ▶). 

Earlier studies have reported beneficial effects of *C. sativus* on blood pressure. The majority of these studies were performed in animal models. The findings of our study were consistent with several studies done in animals. In a study done by Fatehi et al., consumption of 50 mg/100 g *C. sativus* resulted in decreased blood pressure in anaesthetized rats (Fatehi et al., 2003 ▶). The blood pressure lowering effect of *C. sativus* in hypertensive rats may be mediated through blocking calcium channels by crocetin (Imenshahidi et al., 2014 ▶). In one clinical trial conducted on T2D patients, *C. sativus* 15 mg administered twice a day for 8-weeks could not affect the blood pressure, These results are inconsistent with our findings probably due to differences in study duration, and the low dose of *C. sativus* supplementation administered (Milajerdi et al., 2017 ▶). Endothelial dysfunction can play an important role in the pathogenesis of the vascular complications of diabetes and leads to hypertension (De Vriese et al., 2000 ▶). The exact mechanisms explaining the effect of *C. sativus* on blood pressure are unknown, but significant effect of *C. sativus* may be explained by its high content of crocetin that can decrease the expression of soluble intercellular adhesion molecule-1 (sICAM-1) protein and probably decrease arterial stiffness and blood pressure (Xiang et al., 2006 ▶). In Modaghegh et al. study, supplementation with 400 mg *C. sativus* in healthy individuals can reduce SBP during 7 days (Modaghegh et al., 2008 ▶). The results of this study is in line with our results, however, considering the limited evidence of the effect of *C. sativus* consumption on blood pressure, further studies are required to reach a certain conclusion. 

The study results showed that *C. sativus *could not decrease liver enzymes (AST, ALT, and ALP) levels, compared to placebo. In contrast to our findings, in one animal study, 20 mg/kg *C. sativus* had been able to decrease ALT and AST in Wistar rats with fatty liver. These effects of *C. sativus* may be due to modulation of liver enzymes in parallel with major normalization of liver size and structure as well as a decrease of fatty infiltration in hepatocytes (Mashmoul et al., 2016 ▶). In another study, injection of 40 mg/kg *C. sativus *in rats reduced oxidative stress and improved liver and kidney functions (Pitsikas et al., 2008 ▶). These results are inconsistent with our findings probably due to differences in study duration and dose of *C. sativus* used. Milajerdi et al. found that administration of 30 mg/day *C. sativus* extract to T2D patients have no significant effects on ALT, AST, ALP, and nephropathy indices following weeks intervention. These findings are in line with the results of our study (Milajerdi et al., 2017 ▶). More clinical trials using safe dose of *C. sativus* should be performed to approve the present results and find the exact mechanism(s) underlying such effects. 

 Diabetic nephropathy is the usual reason for end-stage kidney disease and structural injury develops over years before clinical and laboratory manifestations such as albuminuria, hypertension, or reduced glomerular filtration rate (GFR), appear (Caramori et al., 2002 ▶; Tavafi et al., 2011 ▶). Although the effect of the *C. sativus* extract on kidney function is rarely investigated in the human studies, a new animal study showed significant reductions of blood urea nitrogen (BUN), and creatinine in the presence of crocin (isolated from *C. sativus*) in diabetic rats (Naghizadeh et al., 2010 ▶). The authors of this study suggested that this kidney protective effect is probably due to the antioxidant effects of crocin. In contrast to our findings, supplementation of high-dose *C. sativus* (400 mg/day) in healthy participants, could increase some kidney hematological indices such as BUN and creatinine in the normal ranges (Modaghegh et al., 2008 ▶). Differences in the dosage of *C. sativus* supplements and health conditions of participants may be reasons for these conflicting results.

As a result of limited funding, we did not measure the serum crocetin levels at the beginning and end of the study. Thus, these limitations should be considered in future studies.

In conclusion, *C. sativus* supplementation in T2D patients had beneficial effects on blood pressure as it decreased SBP after 12 weeks; however, it did not affect serum urea and creatinine, 24-hr urine albumin, liver enzymes, and DBP. Therefore, further clinical trial studies are needed to explain how *C. sativus* affects vascular functions.
